# Relationship between anthropometric indicators and risk factors for cardiovascular disease in adults and older adults of Rio Branco, Acre

**DOI:** 10.11606/s1518-8787.2020054001088

**Published:** 2020-03-09

**Authors:** Nathalia Silva de Lima Loureiro, Thatiana Lameira Maciel Amaral, Cledir de Araújo Amaral, Gina Torres Rego Monteiro, Maurício Teixeira Leite de Vasconcellos, Miguel Junior Sordi Bortolini

**Affiliations:** I Universidade Federal do Acre Programa de Pós-Graduação em Ciências da Saúde Rio Branco AC Brasil Universidade Federal do Acre . Programa de Pós-Graduação em Ciências da Saúde na Amazônia Ocidental. Rio Branco , AC , Brasil; II Universidade Federal do Acre Centro de Ciências da Saúde e do Desporto Programa de Pós-Graduação em Saúde Coletiva Rio Branco AC Brasil Universidade Federal do Acre . Centro de Ciências da Saúde e do Desporto . Programa de Pós-Graduação em Saúde Coletiva . Rio Branco , AC , Brasil; III Instituto Federal de Educação, Ciência e Tecnologia do Acre Campus Rio Branco Rio Branco AC Brasil Instituto Federal de Educação, Ciência e Tecnologia do Acre . Campus Rio Branco . Rio Branco , AC , Brasil; IV Fundação Oswaldo Cruz Escola Nacional de Saúde Pública Sérgio Arouca Rio de Janeiro RJ Brasil Fundação Oswaldo Cruz . Escola Nacional de Saúde Pública Sérgio Arouca . Rio de Janeiro , RJ , Brasil; V Fundação Instituto Brasileiro de Geografia e Estatística Escola Nacional de Ciências Estatísticas Rio de Janeiro RJ Brasil Fundação Instituto Brasileiro de Geografia e Estatística . Escola Nacional de Ciências Estatísticas . Rio de Janeiro , RJ , Brasil; VI Universidade Federal do Acre Centro de Ciências da Saúde e do Desporto Programa de Pós-Graduação em Ciências da Saúde Rio Branco AC Brasil Universidade Federal do Acre . Centro de Ciências da Saúde e do Desporto . Programa de Pós-Graduação em Ciências da Saúde na Amazônia Ocidental. Rio Branco , AC , Brasil

**Keywords:** Cardiovascular diseases, Risk Factors, Body Weights and Measures, Anthropometry, Cross-Sectional Studies

## Abstract

**OBJECTIVE:**

To analyze the association between anthropometric variables and cardiovascular risk factors in adults and older adults of Rio Branco, Acre.

**METHODS:**

A population-based cross-sectional study with 641 adults and 957 older adults was conducted. The statistical analyses consisted of the distribution of anthropometric variables according to the cardiovascular risk factors by frequency and dispersion measures. Pearson’s correlation coefficient and prevalence ratios (PR) were estimated with their respective 95% confidence intervals (95%CI) using the SPSS ^®^ version 20.0.

**RESULTS:**

Moderate correlations were obtained in adult men for waist-hip ratio and total cholesterol (r = 0.486; p < 0.001) and for waist-hip and triglyceride ratios (r = 0.484; p < 0.001). The highest prevalence of hypertension and diabetes in adults were observed in men; in the older adults, the prevalence of hypertension was above 65% in both sexes. The prevalence of dyslipidemia was above 78% in obese adults and older adults. When analyzing the associations, a higher strength of association was found between arterial hypertension and waist-to-stature ratio (PR = 13.42; 95%CI 12.58–14.31) and body mass index greater than 30 kg/m ^2^ (PR = 6.61; 95%CI 6.34–6.89) in adult men. In the analysis of diabetes, the waist-hip ratio presented greater robustness in the association for women (PR = 7.53; 95%CI 6.92–8.20) and men (PR = 9.79; 95%CI 9.14–10.49).

**CONCLUSION:**

Anthropometric variables are important predictors of cardiovascular risk; however, their assessments should be performed independently, according to sex and age group.

## INTRODUCTION

The epidemiological profile of the population has been suffering the influence of chronic non-communicable diseases (CNCD) ^1^ , with obesity emerging as one of the main complications for the development of cardiovascular diseases (CVD) ^2^ , having an influence on its main risk factors, which are arterial hypertension, dyslipidemia, and diabetes ^3^ .

The high prevalence of overweight in developing countries is associated with changes in eating habits and with the sedentary lifestyle ^2^ . According to the World Health Organization (WHO), in 2014, more than 1.9 billion adults were overweight and more than 600 million of these were obese ^5^ , a fact that partly explains CVD as the main causes of death, accounting for 31% of deaths globally ^6^ .

Studies have shown the excess adipose tissue, especially the concentration in the central region of the body, is associated with systemic inflammation, contributing directly to the elevation of cardiovascular morbidity and mortality ^7^ . The atypical presence of visceral fat creates physiological changes that promote lipid changes and may contribute to dyslipidemia, a triggering factor of CVD ^8^ .

Anthropometric indicators, used in assessment of routines on body composition, have been used in the CVD risk prediction because of its practicality, low cost, and good reliability, being widely used both in the clinic and in epidemiological studies ^9^ . However, it remains uncertain which anthropometric variable has greater robustness for the CVD screening. For example, studies indicate that waist circumference (WC) ^10^ and waist-hip ratio (WHR) ^10^ are better for CVD screening than the body mass index (BMI), since they are indicators of fat distribution. But the BMI is still widely used. However, in a study comparing BMI with the conicity index (CI), the former could better predict the CVD incidence and mortality, but they were different for men and women ^11^ . Also in the hypertension and dyslipidemia risk analysis, BMI presented relationships similar to those observed for the WC and WHR variables ^12^ .

This study aims to analyze the association between anthropometric variables and cardiovascular risk factors in the population of adults and older adults, based on the data collected by the Study of Chronic Diseases (EDOC), performed in Rio Branco, Acre.

## METHODS

This article analyzes data from EDOC, a cross-sectional population-based study with adults and the older adults, of both sexes, carried out from April to September 2014 and composed of two household surveys: EDOC-A, with adults (18 to 59 years of age), and EDOC-I, with older adults (60 years of age or older), living in Rio Branco, Acre. Pregnant women and individuals with cognitive impairments that could hinder communication or the understanding of the questions were excluded.

Sampling plans were selected in two stages, census enumeration area (CEA) and households. The first stage was common for EDOC-A and EDOC-I. The CEA were selected with probability proportional to their number and the number of private households in the 2010 Demographic Census (CD2010) of the Brazilian Institute of Geography and Statistics (IBGE). The households were selected by systematic sampling with random starts and distinct ranges per survey. In the selected households for EDOC-A, all living adults were interviewed, and in the selected households for EDOC-I, all older adults were interviewed.

The sample size was estimated considering the 15% prevalence of renal function changes in adults and 40% in older adults ^13^ , with 95% confidence level and 3% absolute error for the simple random sampling. Considering the sampling plan, what is cluster by CEA and household , a 1.95% sampling plan effect was arbitrated to determine the sample size, which received a 20% increase for adults and 12.5% for older adults to compensate the expected non-response rates. This procedure resulted in samples from 652 adults and 1,148 older adults. Dividing these sample sizes by the average number of adults and of older adults per household obtained in CD2010 and defining the selection by CEA of 11 households for EDOC-A and 73 households for EDOC-I, all the sample was obtained from 40 CEA. The effective interviewed sample was 685 adults and 1,020 older adults.

The sample weights were estimated by the inverse of the product of inclusion probabilities in each stage and subsequently calibrated for population data by sex and age groups, using a post-stratification estimator, in order to deal with the typical biases of the home studies and correct non-differential answers ^14^ . The population data used in the calibration of the sample weights were estimated for July 1st, 2014, using the linear trend method that IBGE applies to its population estimates by municipality. For this study, a subsample of the base project was used with 641 adults and 957 older adults who had complete anthropometric measurement. Due to the loss of anthropometric information, it was necessary to perform a new calibration of the sample weights to deal with this non-response (or loss) and obtain weights that produce estimates for 211,902 adults and 23,416 older adults. Further details on the EDOC sampling plan, calculation and calibration of sample weights and different subsamples can be obtained in Amaral et al. 2019 ^15^ .

Anthropometric variables included BMI, WC, WHR, waist-to-stature ratio (WSR), and CI. In all assessments, the protocols recommended by the American College of Sports Medicine ^16^ , all in duplicate, were considered the means of the measurements in each variable.

The weight was measured using a G-Tech Bal Gl 200 digital scale ^®^ with a 50-gram resolution arranged on a flat surface. The interviewees were instructed to wear light clothes and to climb barefoot and with empty pockets in the center of the base of the scale, with the body standing and weight evenly distributed between the two feet, arms beside the body, and looking forward.

The participants’ height was determined by a portable stadiometer Sanny ^®^ , with resolution in millimeters and with the base always arranged on a flat surface. The participant, without using objects on his head, was arranged with his back to the anthropometer, with parallel legs and feet, weight equally distributed in both, arms beside the body, and palms facing the body. After aligning the back of the head, back, buttocks, legs, and heels, and looking forward using the Frankfurt’s plane for head positioning, the interviewee was asked to deeply inspire and hold his breath during the measurement, performed by shifting the moving part of the stadiometer to the highest point of the head, compressing the hair enough to obtain the height measurement.

The BMI was determined by the body mass ratio in kilograms by height square in meters. The following classification for adults was adopted: eutrophic (< 25 kg/m ^2^ ), overweight (25.0 to 29.9 kg/m ^2^ ), and obese (≥ 30 kg/m ^2^ ) ^4^ . For the older adults, the classification was: low weight (< 22 kg/m ^2^ ), eutrophic (22 27 kg/m ^2^ ), and overweight (≥ 27 kg/m ^2^ ) ^17^ .

A Cescorf ^®^ inelastic tape with millimeter resolution was used for the WC measurement, measured at the midpoint between the upper anterior iliac crest and the last rib, with participants breathing normally and with relaxed abdomen. The WC was considered normal when lower than 102 cm in men and lower than 88 cm in women ^4^ . For the hip measurements, the largest region of the gluteal bulge in the horizontal plane was considered, with the participants with their arms slightly in front of their body and feet together. The measurement was read on its side. Both measurements were used to estimate the WHR (WHR = waist/hip measurement), considering adequate values lower than 0.85 for women and less than 0.90 for men ^18^ .

The WSR (WSR = waist/height measurement) was considered adequate when less than 0.5, as recommended by the Brazilian Association for Studies of Obesity and Metabolic Syndrome ^18^ . To estimate the conicity index, the following equation was used:


CI = WC ÷ (0.109 |weight ÷ height)

The blood pressure (BP), expressed in mmHg, was obtained by a blood pressure measurement digital device of model arm BM35 of the Beurer ^®^ brand. The BP was measured three times, one after five minutes of initial rest and two more at two-minute intervals, recording the mean, according to the determinations of the VI Brazilian Guidelines for Hypertension. The systemic arterial hypertension (SAH) was defined as diastolic blood pressure (DBP) ≥ 90 mmHg, systolic blood pressure (SBP) ≥ 140mmHg, and/or current use of anti-hypertensive medication ^20^ .

For laboratory tests of blood samples, peripheral blood was collected from the antecubital fossa, fractionated into two test tubes for triglyceride dosage, total cholesterol, and fractions (HDL: high density lipoprotein, and LDL: low-density lipoprotein), and glycemia, with participants fasting for 12h.

The presence of diabetes was defined according to the criteria of the American Diabetes Association (ADA): ≥ 126 mg/dL fasting plasma glucose or use of oral hypoglycemic or insulin ^21^ . Dyslipidemia was defined by abnormal levels of one or more of the following lipid blood components: ≥ 200 mg/dL triglycerides, ≥ 160 mg/dL total cholesterol, ≥ 150 mg/dL LDL, < 40 mg/dL HDL in men and < 50 mg/dL in women, in addition to the medication record to reduce these values.

The statistical analyses consisted of the distribution of anthropometric variables according to cardiovascular risk factors by frequency and dispersion measures, according to sex and age. To assess the correlation of anthropometric variables with lipid profile, systolic and diastolic blood pressure, and glycemia, the Pearson’s correlation coefficient was used. The level of significance was set at α < 0.05. The association between anthropometric variables and independent variables between men and women was performed with prevalence ratios (PR) and 95% confidence intervals (95%CI). In all analyses, the sample design and weight of observations were considered using the routines of Complex Samples of the Statistical Package SPSS ^®^ version 20.0.

This study was approved by the Research Ethics Committee of the Universidade Federal do Acre, and all the participants signed an informed consent form.

## RESULTS

According to the anthropometric variables, the measures of central tendency of cardiovascular risk factors found that adult men classified with BMI ≥ 30 kg/m ^2^ , WHR ≥ 0.90, WC ≥ 102, WSR > 0.5, and CI > 1.25 presented altered triglyceride averages (TG) and total cholesterol (TC). In women, averages above the TG reference values were observed for all analyzed anthropometric indicators and TC among those with altered conicity index ( Table 1 ).

**Table 1:** Distribution of laboratory test results for cardiovascular risk indicators according to anthropometric variables, by sex, in adults of Rio Branco, Acre. Brazil, 2014.

ADULTS	HDL			LDL			TC			TG			GLI		
	A	SE	Md	A	SE	Md	A	SE	Md	A	SE	Md	A	SE	Md
Men															
BMI (kg/m ^2^ )															
25	49.2	1.44	48.0	98.4	2.85	95.0	168.1	4.27	163.0	109.7	9.90	73.0	91.9	5.67	81.0
25–29.9	45.3	1.30	45.0	124.1	5.19	121.0	195.2	4.82	196.0	177.8	20.0	138.0	91.2	2.74	84.0
≥ 30	43.8	1.82	44.0	115.0	4.81	116.0	203.9	5.30	200.0	233.2	19.3	211.0	99.6	7.06	93.0
CC (cm)															
< 102	47.0	1.10	46.0	109.7	2.05	101.0	181.4	3.28	174.0	145.8	9.80	112.0	91.7	3.26	82.0
≥ 102	46.5	3.23	45.0	114.3	4.59	116.0	207.0	5.61	205.0	246.0	24.9	215.0	104.4	7.71	97.0
RCQ															
< 0.90	45.9	1.11	47.0	102.0	2.46	96.0	169.3	3.08	165.0	108.5	7.18	87.0	86.5	4.60	82.0
≥ 0.90	48.4	2.02	45.0	121.6	4.31	118.0	203.8	4.68	202.0	220.0	16.9	158.0	101.7	4.60	88.0
RCE															
< 0.5	46.1	1.09	96.0	98.0	2.77	94.0	163.8	3.51	160.0	100.5	7.93	76.0	86.8	5.25	81.0
≥ 0.5	47.7	1.83	113.0	121.3	3.61	118.0	201.9	3.69	202.0	204.6	14.4	153.0	98.4	3.86	87.0
IC															
< 1.25	45.6	1.03	44.0	104.2	2.00	99.0	175.0	2.79	170.0	127.5	8.76	108.0	87.8	3.66	82.0
≥ 1.25	50.4	2.86	47.0	125.6	5.29	117.0	200.8	4.70	205.0	225.5	24.6	153.0	105.9	5.71	91.0
Women															
BMI (kg/m ^2^ )															
< 25	51.8	0.87	50.0	105.0	2.61	98.0	176.3	3.24	169.0	104.5	5.95	80.0	81.3	0.93	80.0
25–29.9	51.5	0.90	50.0	112.4	2.66	109.0	191.9	3.35	188.0	145.8	9.63	118.0	86.9	3.84	83.0
≥ 30	49.3	0.90	49.0	114.4	3.18	113.0	192.6	3.73	190.0	151.2	8.44	131.0	90.8	2.29	85.0
CC (cm)															
< 88	51.6	0.54	50.0	107.6	1.84	102.0	181.6	2.24	175.0	114.2	3.98	95.0	81.8	0.85	80.0
≥ 88	50.1	1.13	49.0	115.9	3.36	113.0	196.7	4.53	192.0	171.2	13.5	138.0	94.6	4.91	86.0
RCQ															
< 0.85	51.2	0.63	50.0	107.2	2.13	82.0	180.8	2.73	175.0	113.0	4.69	99.0	82.3	0.89	81.0
≥ 0.85	51.1	1.11	49.0	117.3	3.35	91.0	199.0	4.66	192.0	175.5	14.6	138.0	93.6	5.06	84.0
RCE															
< 0.5	51.6	0.79	50.0	101.4	2.08	96.0	172.9	2.79	168.0	99.2	4.74	83.0	81.2	0.88	80.0
≥ 0.5	50.8	0.71	50.0	117.1	2.32	113.0	196.6	3.05	192.0	156.1	8.19	129.0	88.9	2.69	84.0
IC															
< 1.18	50.8	0.60	49.0	105.7	1.81	102.0	175.0	2.79	173.0	109.5	3.49	95.0	81.9	0.94	80.0
≥ 1.18	51.9	1.00	51.0	118.5	3.43	113.0	206.2	5.75	192.0	171.6	12.99	138.0	92.3	4.26	85.0

BMI: body mass index; WC: waist circumference; WHR: waist-hip ratio; WSR: waist-to-stature ratio; CI: conicity index; HDL: high-density lipoprotein; LDL: low-density lipoprotein; TG: triglycerides; TC: total cholesterol; GLI: glycemia; SE: Standard Error; A: average; Md: median

Among older adults, triglyceride levels were elevated in individuals with BMI, WC, WHR, WSR and both sexes showed altered CI. Among the women, higher TC mean values were observed in all altered indicators ( Table 2 ).

**Table 2:** Distribution of laboratory test results for cardiovascular risk indicators according to anthropometric variables, by sex, in older adults of Rio Branco, Acre. Brazil, 2014.

OLDER ADULTS	HDL			LDL			TC			TG			GLI		
	A	SE	Md	A	SE	Md	A	SE	Md	A	SE	Md	A	SE	Md
Men															
BMI (kg/m ^2^ )															
< 22	49.5	1.28	51.0	109.3	3.63	109.0	180.3	5.04	175.0	108.1	9.01	85.0	96.4	6.41	83.0
22–27	49.0	0.86	48.0	120.1	2.88	115.0	197.1	3.56	193.0	147.8	7.58	116.0	95.7	3.92	84.0
> 27	46.8	1.55	44.0	116.0	3.31	118.0	197.6	3.27	195.0	196.7	10.6	165.0	101.4	3.02	91.0
CC (cm)															
< 102	48.2	0.69	47.0	116.4	2.18	115.0	194.4	2.75	191.0	157.3	7.03	125.0	97.8	2.87	86.0
≥ 102	47.6	3.29	43.0	119.7	5.75	119.0	199.2	4.98	204.0	197.2	12.0	184.0	100.4	2.30	96.0
RCQ															
< 1.0	51.3	1.40	51.0	118.7	5.84	119.0	193.6	6.70	193.0	122.0	9.93	92.0	102.4	6.30	86.0
≥ 1.0	47.6	1.02	46.0	117.0	2.07	115.0	195.7	2.48	192.0	171.0	7.97	140.0	98.0	2.79	87.0
RCE															
< 0.5	50.7	1.41	51.0	109.1	4.03	109.0	181.9	5.62	179.0	111.4	9.50	94.0	101.2	6.26	84.0
≥ 0.5	47.7	0.92	46.0	118.2	2.08	117.0	197.1	2.41	195.0	171.5	7.58	140.0	97.9	2.80	87.0
IC															
< 1.25	49.7	1.12	48.0	115.4	3.45	117.0	191.4	4.38	187.0	134.5	8.61	110.0	98.9	3.80	86.0
≥ 1.25	47.5	1.04	46.0	117.5	2.23	115.0	196.7	2.58	194.0	175.2	8.48	143.0	98.0	2.92	97.0
Women															
BMI (kg/m ^2^ )															
< 22	56.9	1.62	57.0	123.3	4.09	122.0	205.7	5.53	210.0	131.5	7.13	116.0	96.8	8.08	86.0
22–27	58.0	1.26	56.0	131.1	3.28	127.0	221.0	4.52	218.0	161.2	6.57	136.0	104.7	4.20	88.0
> 27	54.3	0.88	53.0	124.1	2.56	121.0	212.8	3.07	205.0	179.2	5.68	158.0	104.6	3.00	92.0
CC (cm)															
< 88	57.3	0.88	56.0	129.7	2.79	127.0	216.8	3.60	213.0	154.3	4.78	134.0	101.2	3.70	87.0
≥ 88	54.5	0.95	53.0	123.0	2.26	119.0	212.6	2.84	205.0	179.7	6.00	158.0	105.7	3.45	92.0
RCQ															
< 0.85	58.2	1.26	57.0	125.9	3.45	121.0	213.1	4.61	205.0	149.6	6.46	135.0	98.9	4.50	87.0
≥ 0.85	55.0	0.77	54.0	126.6	2.04	124.0	215.2	2.45	211.0	173.4	4.66	154.0	105.1	2.94	90.0
RCE															
< 0.5	57.8	1.94	57.0	125.6	5.39	122.0	211.1	6.75	210.0	135.0	6.45	120.0	103.3	9.84	87.0
≥ 0.5	55.6	0.67	55.0	126.5	1.88	123.0	215.2	2.35	210.0	171.5	4.39	151.0	103.4	2.45	90.0
IC															
< 1.18	57.9	1.09	55.0	129.1	3.91	123.0	216.6	5.49	211.0	155.0	8.50	135.0	101.9	6.54	87.0
≥ 1.18	55.4	0.72	55.0	125.7	2.01	122.0	214.2	2.40	210.0	169.8	3.93	150.0	103.8	2.57	90.0

BMI: body mass index; WC: waist circumference; WHR: waist-hip ratio; WSR: waist-to-stature ratio; CI: conicity index; HDL: high-density lipoprotein; LDL: low-density lipoprotein; TG: triglycerides; TC: total cholesterol; GLI: glycemia; SE: Standard Error; A: average; Md: median

The highest frequencies of overweight and obesity in adults, according to BMI, were observed in the age group from 40 to 59 years: 39.5% and 22.6% for men and 44.4% and 31.5% for women. Regarding central obesity, verified by WC, WHR, and WSR indices, higher rates were also identified in the age group from 40 to 59 years, in both sexes ( Table 3 ).

**Table 3:** Distribution of anthropometric variables, according to age and sex, in adults and older adults of Rio Branco, Acre. Brazil, 2014.

	ADULTS							OLDER ADULTS					
	18–39 years old			40–59 years old				60–79 years old			80 years old or older		
	n	N	%	n	N	%		n	N	%	n	N	%
BMI (kg/m ^2^ )							BMI (kg/m ^2^ )						
Men							Men						
< 25	48	36,327	51.6	38	11,821	37.9	< 22	31	879	9.4	19	401	26.0
25–29.9	37	25,405	36.0	38	12,318	39.5	22–27	148	4,256	45.5	33	695	45.0
≥ 30	13	8,711	12.4	22	7,041	22.6	> 27	140	4,216	45.1	21	448	29.0
Women							Women						
< 25	119	37,247	49.0	51	8,248	24.1	< 22	49	1,128	10.5	28	542	30.3
25–29.9	80	25,094	33.0	88	15,221	44.4	22–27	160	3,588	33.5	27	523	29.2
≥ 30	44	13,663	18.0	63	10,805	31.5	> 27	263	6,013	56.0	38	726	40.5
WC (cm)							WC (cm)						
Men							Men						
< 102	93	67,384	95.7	80	25,280	81.1	< 102	254	7,411	79.6	61	1,291	84.8
≥102	05	3,059	4.3	18	5,901	18.9	≥102	64	1,899	20.4	11	232	15.2
Women							Women						
< 88	190	59,501	78.6	114	19,190	56.0	< 88	213	4,851	45.4	52	1,003	56.0
≥ 88	52	16,197	21.4	88	15,084	44.0	≥ 88	257	5,832	54.6	41	788	44.0
WHR							WHR						
Men							Men						
< 0.90	66	49,096	69.7	31	9,865	31.6	< 0.90	38	1,098	11.9	10	216	14.2
≥ 0.90	32	21,347	30.3	67	21,316	68.4	≥ 0.90	278	8,155	88.1	62	1,307	85.8
Women							Women						
< 0.85	199	62,699	82.3	100	17,697	51.6	< 0.85	136	3,131	29.4	19	363	20.5
≥ 0.85	43	13,399	17.7	102	16,577	48.4	≥ 0.85	333	7,528	70.6	73	1,406	79.5
WSR							WSR						
Men							Men						
< 0.5	56	42,657	60.6	19	5,946	19.1	< 0.5	38	1,108	11.9	07	150	9.9
< 0.5	42	27,786	39.4	79	25,235	80.9	< 0.5	280	8,202	88.1	65	1,372	90.1
Women							Women						
< 0.5	134	42,082	55.6	47	8,108	23.7	< 0.5	56	1,292	12.1	10	190	10.6
≥ 0.5	108	33,616	44.4	155	26,166	76.3	> 0.5	414	9,391	87.9	83	1,601	89.4
CI							CI						
Men							Men						
< 1.25	81	59,007	83.8	44	14,493	46.5	< 1.25	87	2,614	28.1	11	238	15.7
≥ 1.25	17	11,436	16.2	54	16,688	53.5	≥ 1.25	231	6,696	71.9	61	1,284	84.3
Women							Women						
< 1.18	191	59,715	78.9	80	13,892	40.5	< 1.18	96	2,224	20.8	7	134	7.5
≥ 1.18	51	15,983	21.1	122	20,382	59.5	≥ 1.18	374	8,459	79.2	86	1,657	92.5

BMI: body mass index; WC: waist circumference; WHR: waist-hip ratio; WSR: waist-to-stature ratio; CI: Conicity index; N:n expanded to the population.

Among older adults, the highest frequencies of overweight due to BMI were observed in the age group from 60 to 79 years (45.1% for men and 56.0% for women). In women aged 80 years or older, a high frequency of overweight was also observed. When analyzing central obesity in this same population according to WHR and WSR, frequencies above 70% were obtained in both age groups in both sexes ( Table 3 ).

Anthropometric variables showed statistically significant correlations with lipid profile variables and glycemia; however, they were moderate correlations, and the most expressive were in men with WHR, WC, and WSR correlation coefficients with triglyceride (r = 0.484, r = 0.438, and r = 0.448, respectively) and with total cholesterol (r = 0.486, r = 0.445, and r = 0.475, respectively). In adult women, the highest correlations observed were WHR with total cholesterol (r = 0.350) and triglycerides (r = 0.345), and WSR with SBP (r = 0.369). For older adults, the highest statistically significant correlation was observed in men, between triglycerides and BMI (r = 0.251), as shown in Table 4 .

**Table 4:** Correlation matrix between anthropometric variables, lipid profile, glycemia, and blood pressure according to sex in adults and in older adults of Rio Branco, Acre. Brazil, 2014.

	HDL	LDL	TG	TC	GLI	SBP	DBP
ADULTS							
Women							
BMI	-0.112 ^a^	0.100 ^a^	0.259 ^a^	0.147 ^a^	0.161 ^a^	0.288 ^a^	0.252 ^a^
WC	-0.079 ^a^	0.141 ^a^	0.33 ^6^ a	0.204 ^a^	0.210 ^a^	0.321 ^a^	0.275 ^a^
WHR	-0.040 ^a^	0.115 ^a^	0.345 ^a^	0.35 ^0^ a	0.246 ^a^	0.337 ^a^	0.316 ^a^
WSR	-0.043 ^b^	0.149 ^b^	0.336	0.226 ^b^	0.196 ^b^	0.369 ^b^	0.321 ^a^
CI	-0.026 ^a^	0.005	-0.022 ^a^	-0.010 ^a^	-0.007 ^b^	-0.003	0.031 ^a^
Men							
BMI	-0.145 ^a^	0.241 ^a^	0.37 ^7^ a	0.36 ^8^ a	-0.022 ^a^	0.243 ^a^	0.203 ^a^
WC	-0.026 ^a^	0.269 ^a^	0.43 ^8^ a	0.44 ^5^ a	0.047 ^a^	0.401 ^a^	0.350 ^a^
WHR	0.027 ^a^	0.286 ^a^	0.48 ^4^ a	0.48 ^6^ a	0.142 ^a^	0.340 ^a^	0.371 ^b^
WSR	-0.036 ^a^	0.300 ^a^	0.448 ^a^	0.475 ^a^	0.096 ^a^	0.377 ^a^	0.351 ^a^
CI	0.135 ^a^	0.201 ^a^	0.324 ^a^	0.369 ^a^	0.161 ^a^	0.422 ^a^	0.404 ^a^
OLDER ADULTS							
Women							
BMI	-0.110 ^a^	-0.044 ^a^	0.102 ^a^	-0.021 ^b^	0.034 ^a^	0.051 ^a^	0.140 ^a^
WC	-0.039 ^a^	-0.037 ^a^	-0.038 ^a^	-0.055 ^a^	0.051 ^a^	0.064 ^a^	-0.010
WHR	-0.149 ^a^	-0.024 ^a^	0.174 ^a^	0.077 ^a^	0.077 ^a^	0.124 ^a^	0.151 ^a^
WSR	-0.129 ^b^	-0.079 ^a^	0.114 ^b^	-0.044 ^a^	0.053 ^a^	0.109 ^a^	0.176 ^a^
CI	-0.038 ^a^	-0.037 ^a^	-0.039 ^a^	-0.055 ^a^	0.051 ^a^	0.064 ^a^	-0.010
Men							
BMI	-0.088 ^a^	0.056 ^a^	0.251 ^a^	-0.110 ^a^	0.052 ^a^	0.174 ^a^	0.162 ^a^
WC	0.056 ^a^	0.028 ^a^	-0.051 ^a^	0.020 ^a^	-0.029 ^a^	-0.014	-0.025 ^a^
WHR	-0.063 ^a^	0.005	0.073 ^a^	-0.007	0.000	0.130 ^a^	0.068 ^a^
WSR	-0.082 ^a^	0.050 ^ba^	0.219 ^a^	0.089 ^a^	0.034 ^a^	0.148 ^a^	0.156 ^a^
CI	0.056 ^a^	0.027 ^a^	-0.052 ^a^	0.019 ^b^	-0.029 ^a^	-0.014	-0.025 ^a^

BMI: body mass index; WC: waist circumference; WHR: waist-hip ratio; WSR: waist-to-stature ratio; CI: conicity index; HDL: high-density lipoprotein; LDL: low-density lipoprotein; TG: triglycerides; TC: total cholesterol; GLI: glycemia; SBP: systolic blood pressure; DBP: diastolic blood pressure

^a^ p<0.01

^b^ p<0.05

The prevalence of hypertension was higher in obese men according to all analyzed indicators. Also, the highest prevalence of diabetes was observed in men, the highest being the CI anthropometric indicator (16.3%). In adults of both sexes, the prevalence above 78% of dyslipidemia in obese individuals was highlighted according to all analyzed indicators ( Table 5 ).

**Table 5:** Association between anthropometric variables and cardiovascular risk factors according to sex, in adults of Rio Branco, Acre. Brazil, 2014.

	ADULTS	HYPERTENSION		DIABETES		DYSLIPIDEMIA	
		Prevalence (%)	PR (95%CI)	Prevalence (%)	PR (95%CI)	Prevalence (%)	PR (95%CI)
Women							
BMI (kg/m ^2^ )							
	25–29.9	14.6	2.32 (2.22–2.43)	2.2	2.34 (2.09–2.63)	78.7	1.16 (1.14–1.18)
	≥ 30	15.4	2.46 (2.34–2.58)	6.1	6.50 (5.84–7.24)	82.5	1.22 (1.20–1.24)
WC (cm)	≥ 88	19.8	2.51 (2.42–2.60)	6.6	7.10 (6.52–7.72)	81.8	1.13 (1.11–1.15)
WHR	≥ 0.85	20.6	2.65 (2.56–2.74)	6.8	7.53 (6.92–8.20)	84.1	1.17 (1.15–1.19)
WSR	> 0.5	15.5	2.51 (2.41–2.62)	3.9	4.63 (4.17–5.13)	80.8	1.18 (1.16–1.20)
CI	≥ 1.18	20.3	2.96 (2.85–3.07)	6.0	7.39 (6.75–8.10)	81.1	1.12 (1.11–1.14)
Men							
BMI (kg/m ^2^ )	25–29.9	14.8	2.19 (2.09–2.29)	8.1	1.47 (1.40–1.55)	78.1	2.04 (2.01–2.08)
	≥ 30	44.8	6.61 (6.34–6.89)	10.6	1.92 (1.81–2.04)	82.3	2.15 (2.11–2.20)
WC (cm)	≥ 102	63.3	5.74 (5.56–5.93)	8.9	1.27 (1.17–1.36)	80.9	1.39 (1.36–1.43)
WHR	≥ 0.90	30.7	6.08 (5.84–6.32)	15.3	9.79 (9.14–10.49)	80.7	1.78 (1.75–1.81)
WSR	> 0.5	28.1	13.42 (12.58–14.31)	12.2	6.41 (5.98–6.87)	79.4	2.03 (1.99–2.06)
CI	≥ 1.25	37.3	5.10 (4.93–5.27)	16.3	4.32 (4.12–4.53)	80.0	1.52 (1.49–1.54)
Total							
BMI (kg/m ^2^ )	25–29.9	14.7	2.25 (2.18–2.32)	5.1	1.55 (1.47–1.62)	78.4	1.49 (1.47–1.51)
	≥ 30	27.0	4.14 (4.01–4.27)	7.9	2.40 (2.28–2.52)	82.4	1.57 (1.54–1.59)
WC (cm)	≥ 88 ≥ 102	29.5	3.01 (3.01–3.15)	7.1	1.67 (1.60–1.74)	81.6	1.26 (1.25–1.28)
WHR	≥ 0.85 ≥ 0.90	26.5	4.00 (3.90–4.10)	11.8	9.92 (9.41–10.46)	82.1	1.36 (1.34–1.37)
WSR	> 0.5	21.4	5.13 (4.96–5.30)	7.8	5.71 (5.39–6.04)	80.1	1.48 (1.47–1.50)
CI	≥ 1.18 ≥ 1.25	27.8	3.92 (3.82–4.01)	10.5	4.57 (4.38–4.76)	80.6	1.29 (1.28–1.31)
Women							
BMI (kg/m ^2^ )	> 27	71.5	1.65 (1.61–1.69)	20.8	1.15 (1.06–1.09)	87.3	1.05 (1.03–1.07)
WC (cm)	≥ 88	70.6	1.15 (1.13–1.66)	20.1	1.08 (1.06–1.09)	87.8	1.05 (1.03–1.06)
WHR	≥ 0.85	68.3	1.16 (1.14–1.18)	18.2	1.06 (1.05–1.08)	88.7	1.12 (1.01–1.13)
WSR	> 0.5	66.5	1.21 (1.18–1.24)	17.4	1.07 (1.05–1.09)	86.0	1.02 (1.00–1.04)
CI	≥ 1.18	65.4	1.07 (1.04–1.09)	17.5	1.05 (1.03–1.07)	86.8	1.06 (1.04–1.07)
Men							
BMI (kg/m ^2^ )	> 27	77.8	1.57 (1.53–1.61)	19.2	1.12 (1.10–1.15)	83.0	1.42 (1.38–1.46)
WC (cm)	≥ 102	72.1	1.09 (1.06–1.11)	22.6	1.11 (1.09–1.13)	89.1	1.23 (1.20–1.25)
WHR	≥ 0.90	67.7	1.18 (1.15–1.21)	14.2	0.99 (0.97–1.01)	75.7	1.23 (1.20–1.26)
WSR	> 0.5	67.4	1.18 (1.15–1.21)	14.5	1.03 (1.01–1.05)	75.9	1.32 (1.28–1.35)
CI	≥ 1.25	67.7	1.08 (1.06–1.11)	14.2	1.00 (0.99–1.02)	77.0	1.18 (1.16–1.20)
Total							
BMI (kg/m ^2^ )	> 27	74.1	1.30 (1.59–1.64)	20.2	1.14 (1.12–1.15)	85.5	1.13 (1.12–1.15)
WC (cm)	≥ 88 ≥ 102	70.9	1.10 (1.09–1.12)	20.7	1.09 (1.08–1.10)	88.1	1.15 (1.13–1.16)
WHR	≥ 0.85 ≥ 0.90	68.0	1.16 (1.15–1.18)	16.2	1.03 (1.02–1.04)	82.0	1.11 (1.10–1.13)
WSR	> 0.5	64.8	1.20 (1.17–1.22)	16.1	1.05 (1.03–1.06)	81.3	1.15 (1.13–1.16)
CI	≥ 1.18 ≥ 1.25	66.4	1.07 (1.06–1.09)	16.1	1.03 (1.01–1.04)	82.5	1.20 (1.18–1.22)

BMI: body mass index; WC: waist circumference; WHR: waist-hip ratio; WSR: waist-to-stature ratio; CI: conicity index; PR: prevalence ratio; 95%CI: 95% confidence interval

When analyzing the associations, a higher strength of association was found between hypertension and WSR (PR = 13.42; 95%CI 12.58–14.31) and with BMI > 30 (PR = 6.61; 95%CI 6.34–6.89) in adult men. In the analysis of diabetes, the WHR presented greater robustness in the association for women (PR = 7.53; 95%CI 6.92–8.20) and men (PR = 9.79; 95%CI 9.14–10.49), as shown in Table 5 .

Among the older adults, the prevalence of hypertension and obesity was higher than that found in the adult group. The prevalence of hypertension was above 65% for men and women. For diabetes, the highest prevalence was 20.8% among those with BMI > 27 in women and 22.6% for altered WC in men. The prevalence of dyslipidemia was similar to that observed in adults ( Table 5 ).

In the bivariate analysis, among the older adults, there were lower effects of association between anthropometric indicators and outcomes than among adults. The highest association observed was between BMI and hypertension (PR = 1.65; 95%CI 1.61–1.69) in older men, although the prevalence ratios remained similar in both sexes in this age group ( Table 5 ).

## DISCUSSION

Among the main findings of this study, the correlations between WHR, WC, and WSR with TG and TC in adult men are highlighted. The highest frequencies of general obesity were found in adults aged 40 to 59 years and in older adults aged 60 to 69 years. The lipid profile markers were elevated in adults of both sexes with BMI, WC, WHR, WSR, and altered CI.

Epidemiological studies have shown a clear correlation between obesity and cardiovascular risk factors ^22 , 23^ . In this analysis, according to anthropometric classification indices, overweight and obesity were frequent. The Surveillance System for Risk and Protective Factors for Chronic Diseases by Telephone Survey showed that 53.8% of the Brazilian population over 18 years of age had some degree of overweight, with Rio Branco being the capital with the highest prevalence (60.6%) ^24^ .

International ^2 , 25^ and Brazilian ^23 , 24^ studies showed a high prevalence of overweight, a worldwide phenomenon known as a nutritional transition that has changes in the dietary pattern and physical activity as determining factors ^2 , 4^ . Currently it is a worldwide epidemic, which affects all age groups, different socioeconomic groups, and countries, causing numerous injuries and making this nutritional disorder harmful to public health ^4^ .

In this investigation, the total cholesterol (TC) and triglycerides (TG) were more correlated with WC, WHR, and WSR in men. Similar results were found in an Iranian study, which found correlations of TC and TG with most anthropometric indices, especially in men ^22^ .

Although BMI is usually used in obesity screening, abdominal measurements are being widely employed in predicting risk factors for CVD ^12^ . In part, this stems from the observation that abdominal fat is related to various metabolic abnormalities, including adversities of the lipid profile ^12^ .

In this study, the prevalence of dyslipidemia was above 75%, regardless of sex, age, and anthropometric indicator analyzed. In a population-based study performed in São Paulo, the prevalence of dyslipidemias was 73.1% and 69.9% in adults and in older adults, respectively, overweight, according to BMI, and 70.4% and 64.2% according to the increased WC ^26^ . Another population-based study with older adults from southern Brazil showed that 70% of obese women had hypercholesterolemia and 64%, hypertriglyceridemia; on the other hand, in men, the prevalence was 38.9% and 50.0%, respectively ^23^ .

Obese individuals are more susceptible to develop diabetes and, especially when obesity is centered in the abdominal region, negative repercussions are more expressive ^10^ , both metabolic and cardiovascular. Since visceral fat is pro-inflammatory, it can be infiltrated by macrophages that can lead to endothelial dysfunction and subsequent insulin resistance ^7^ .

A study with the older population in São Paulo identified a prevalence of 21.3% of diabetes among those with BMI > 27, similar to that observed in this study: 20.2% in the older population ^27^ . Although older adults had higher prevalence regarding that observed among adults, the probability ratios were stronger among adults. A possible explanation would rest on the survival bias, since those more vulnerable to complications caused by the disease would be more likely to die prematurely ^28^ .

In adults, the WHR indicator had the strongest association with diabetes in both sexes. A review study identified WHR and WC as the best predictors of risk factors for cardiovascular disease ^10^ .

In this study, the highest frequencies of general and central obesity in older adults were observed in the range of 60 to 79 years in both sexes. Current tendencies indicate the prevalence in this range will increase, even among older groups. In the Scottish Health Survey, performed between 1998 and 2008, the obesity verified by BMI continued to increase even among individuals aged 60 to 70 years. In the same period, an increase of 5 to 10 cm of WC in both sexes in the range of 50 to 70 years was observed ^25^ .

Overweight is associated with arterial hypertension ^20 , 29^ . This fact can be explained by physiological alterations such as activation of the sympathetic nervous system and the renin-angiotensin-aldosterone system, insulin resistance, and renal and endothelial dysfunction ^30^ .

In this study, the higher prevalence of hypertension was observed more in obese men (44.8%) than in obese women (15.4%) according to BMI. Similar data were found in a population-based study conducted in São Luís (MA), in which 32.1% of men and 24.2% of women were hypertensive ^29^ . The VI Brazilian Guidelines for Hypertension indicate that the overall prevalence of hypertension among men is higher in men up to 50 years of age, reversing after the fifth decade ^20^ .

Obesity was a risk factor for arterial hypertension, because obese individuals of both sexes, according to BMI and WSR, had an increase of 6 to 13 times the risk of having hypertension, respectively. A proportional relationship was also observed between the prevalence of hypertension and the increase in WC and WHR, especially in men. In a study with individuals over 18 years of age, BMI and WC indicators were considered good predictors of the risk for developing hypertension ^29^ .

In older adults, the prevalence of hypertension above 65% was found in men and women with overweight and obesity. Aging was associated with an increase in the prevalence of systemic arterial hypertension (SAH) ^29^ due to the distensibility of the aorta (complacency), reduction in the systolic volume of the left ventricle, and the ejection velocity of the left ventricle ^31^ .

Some limitations can be recognized in this study. In the analysis of the results of the older adults, caution is necessary due to survival bias, considering that risk factors for cardiovascular diseases lead to early death in the older adults affected by them. Other longitudinal analyses are needed to provide stronger evidence of the relationships obtained in this study.

It is also important to highlight that the collection of biological samples was performed at a single moment in time to define morbidities. However, all analyses were performed in the same laboratory to minimize errors, and it is important to use these results to obtain greater reliability in the definitions of cardiovascular risk factors. Furthermore, possible errors in the verification of anthropometric measurements were minimized by duplicity in the verification and use of means. It is also highlighted as one of the strengths of this study the fact of working with a population-based sample representative of adults and older adults in the Rio Branco.

The obtained results show the relevance of these indicators in the identification of CVD risk factors and the importance of adopting them in clinical practice and epidemiological studies with adults and older adults, considering that they are simple, low-cost, and noninvasive methods. These indicators can contribute to the early identification of risk factors, enabling actions and strategies to prevent and control cardiovascular diseases.
