# Supplementary material for: Relationship between anthropometric indicators and risk factors for cardiovascular disease in adults and older adults of Rio Branco, Acre
Source: Rev Saude Publica. 2020 Mar 9;54:24. doi: 10.11606/s1518-8787.2020054001088 (PMC9586447; doi:10.11606/s1518-8787.2020054001088)
Supplement: Translation

# Translation: Relação de indicadores antropométricos com fatores de risco para doença cardiovascular em adultos e idosos de Rio Branco, Acre

**Authors:** Nathalia Silva de Lima Loureiro, Thatiana Lameira Maciel Amaral, Cledir de Araújo Amaral, Gina Torres Rego Monteiro, Maurício Teixeira Leite de Vasconcellos, Miguel Junior Sordi Bortolini

**Affiliations:** I Brasil Universidade Federal do Acre. Programa de Pós-Graduação em Ciências da Saúde na Amazônia Ocidental. Rio Branco, AC, Brasil; II Brasil Universidade Federal do Acre. Centro de Ciências da Saúde e do Desporto. Programa de Pós-Graduação em Saúde Coletiva. Rio Branco, AC, Brasil; III Brasil Instituto Federal de Educação, Ciência e Tecnologia do Acre. Campus Rio Branco. Rio Branco, AC, Brasil; IV Brasil Fundação Oswaldo Cruz. Escola Nacional de Saúde Pública Sérgio Arouca. Rio de Janeiro, RJ, Brasil; V Brasil Fundação Instituto Brasileiro de Geografia e Estatística. Escola Nacional de Ciências Estatísticas. Rio de Janeiro, RJ, Brasil; VI Brasil Universidade Federal do Acre. Centro de Ciências da Saúde e do Desporto. Programa de Pós-Graduação em Ciências da Saúde na Amazônia Ocidental. Rio Branco, AC, Brasil

**Keywords:** Doenças Cardiovasculares, Fatores de Risco, Pesos e Medidas Corporais, Antropometria, Estudos Transversais

## Abstract

**OBJETIVO:**

Analisar a associação entre variáveis antropométricas e os fatores de risco cardiovascular na população de adultos e idosos de Rio Branco, Acre.

**MÉTODOS:**

Estudo transversal de base populacional com 641 adultos e 957 idosos. As análises estatísticas consistiram na distribuição das variáveis antropométricas segundo os fatores de risco cardiovascular por medidas de frequência e dispersão. Foram calculadas a correlação de Pearson e razões de prevalência (RP) com seus respectivos intervalos de confiança de 95% (IC5%), empregando as rotinas do SPSS ^®^ versão 20.0.

**RESULTADOS:**

Correlações moderadas foram obtidas nos adultos homens para relação cintura-quadril e colesterol total (r = 0,486; p < 0,001) e para relação cintura-quadril e triglicerídeos (r = 0,484; p < 0,001). As maiores prevalências de hipertensão arterial e diabetes nos adultos foram observadas nos homens; já nos idosos, as prevalências de hipertensão ficaram acima de 65% em ambos os sexos. As prevalências de dislipidemia ficaram acima de 78% nos indivíduos obesos adultos e idosos. Ao analisar as associações, constatou-se maior força de associação entre hipertensão arterial e relação cintura-estatura (RP = 13,42; IC95% 12,58–14,31) e com índice de massa corporal maior que 30 kg/m ^2^ (RP = 6,61; IC95% 6,34–6,89) nos homens adultos. Na análise para diabetes, a relação cintura-quadril apresentou maior robustez na associação para mulheres (RP = 7,53; IC95% 6,92–8,20) e homens (RP = 9,79; IC95% 9,14–10,49).

**CONCLUSÃO:**

As variáveis antropométricas são importantes preditores de risco cardiovascular; no entanto, suas avaliações devem ser feitas de forma independente, segundo sexo e grupo etário.

## INTRODUÇÃO

O perfil epidemiológico da população vem sofrendo a influência das doenças crônicas não transmissíveis (DCNT) ^1^ , com a obesidade destacando-se como um dos principais complicadores para o desenvolvimento das doenças cardiovasculares (DCV) ^2^ , tendo influência nos seus principais fatores de risco, que são a hipertensão arterial, a dislipidemia e o diabetes ^3^ .

A alta prevalência de excesso de peso em países em desenvolvimento está associada a mudanças nos hábitos alimentares e ao sedentarismo ^2^ . De acordo com a Organização Mundial de Saúde (OMS), em 2014, mais de 1,9 bilhões de adultos tinham excesso de peso mais de 600 milhões desses eram obesos ^5^ , fato que explica, em parte, as DCV como as principais causas de morte, responsáveis por 31% dos óbitos em nível global ^6^ .

Estudos têm demonstrado que o excesso de tecido adiposo, principalmente a concentração na região central do corpo, está associado à inflamação sistêmica, contribuindo diretamente para a elevação da morbimortalidade cardiovascular ^7^ . A presença atípica de gordura visceral gera modificações fisiológicas que promovem alterações lipídicas, podendo corroborar para o quadro de dislipidemia, fator desencadeante das DCV ^8^ .

Indicadores antropométricos, empregados nas rotinas de avaliação da composição corporal, têm sido utilizados na predição de risco de DCV pela praticidade, baixo custo e boa confiabilidade, sendo amplamente empregados tanto na clínica como em estudos epidemiológicos ^9^ . No entanto, permanece incerto qual variável antropométrica possui maior robustez para o rastreamento das DCV. A título de exemplo, estudos apontam que a circunferência da cintura (CC) ^10^ e relação cintura-quadril (RCQ) ^10^ são melhores para o rastreamento das DCV, uma vez que são indicadores da distribuição da gordura, do que o índice de massa corporal (IMC), mas este continua sendo amplamente utilizado. No entanto, em estudo comparando o IMC ao índice de conicidade (IC), o primeiro demostrou prever melhor a incidência e a mortalidade por DCV, porém de forma diferente para homens e mulheres ^11^ . Também na análise do risco de hipertensão arterial e dislipidemia, o IMC apresentou relações semelhantes às observadas para as variáveis CC e RCQ ^12^ .

O presente estudo tem por objetivo analisar a associação entre variáveis antropométricas e os fatores de risco cardiovascular na população de adultos e idosos com base nos dados coletados pelo Estudo das Doenças Crônicas (Edoc), realizado em Rio Branco, Acre.

## MÉTODOS

Os dados provêm do Edoc, um estudo transversal de base populacional com adultos e idosos de ambos os sexos realizado no período de abril a setembro de 2014 e composto por duas pesquisas domiciliares: EDOC-A, sobre adultos (18 a 59 anos), e EDOC-I, sobre idosos (60 anos ou mais), residentes em Rio Branco, Acre. Foram excluídos da população de pesquisa as mulheres grávidas e os indivíduos com comprometimentos cognitivos que inviabilizassem a comunicação ou o entendimento das perguntas.

Os planos de amostragem foram selecionados em dois estágios, setor e domicílio, sendo o primeiro estágio comum às duas pesquisas. A seleção dos setores foi feita com probabilidade proporcional ao seu número e domicílios particulares no Censo Demográfico 2010 (CD2010), do Instituto Brasileiro de Geografia e Estatística (IBGE). Os domicílios foram selecionados por amostragem sistemática com inícios aleatórios e intervalos distintos por pesquisa. Nos domicílios selecionados para o Edoc-A, todos os adultos moradores foram entrevistados, assim como nos domicílios selecionados para o Edoc-I, todos os idosos foram entrevistados.

O tamanho das amostras foi calculado considerando as prevalências de alteração da função renal de 15% em adultos e de 40% em idosos ^13^ , com grau de confiança de 95% e erro absoluto de 3% para amostragem aleatória simples de proporções. Considerando que o plano de amostragem é conglomerado por setor, foi arbitrado um efeito de plano de amostragem de 1,95 para determinar os tamanhos das amostras, que receberam acréscimos de 20% para o Edoc-A e 12,5% para o Edoc-I para compensar as não respostas esperadas. Esse procedimento resultou em amostras de 652 adultos e 1.148 idosos. Dividindo esses tamanhos de amostra pelo número médio de adultos e de idosos por domicílio, obtidos no CD2010 e definindo a seleção, por setor, de 11 domicílios para o Edoc-A e 73 domicílios para o Edoc-I, foi obtido um tamanho para a amostra de setores de 40. A amostra efetiva foi de 685 adultos e 1.020 idosos entrevistados.

Os pesos amostrais foram calculados pelo inverso das probabilidades de inclusão em cada estágio e posteriormente calibrados para dados populacionais por sexo e grupos de idade, usando um estimador de pós-estratificação, de forma a lidar com os vieses típicos das pesquisas domiciliares e corrigir não respostas diferenciais ^14^ .Os dados populacionais usados na calibração dos pesos amostrais foram estimados para 1º de julho de 2014, usando o método da tendência linear que o IBGE aplica em suas estimativas populacionais por município. Para o presente estudo, foi usada uma subamostra do projeto-base, com 641 adultos e 957 idosos, que tiveram medição antropométrica completa. Em decorrência da perda de informação antropométrica, foi necessário proceder a uma nova calibração dos pesos amostrais para lidar com essa não resposta (ou perda) e obter pesos que produzam estimativas para 211.902 adultos e 23.416 idosos. Maiores detalhes sobre o plano de amostragem do Edoc, cálculo e calibração dos pesos da amostra e de distintas subamostras podem ser obtidos no artigo de Amaral et al. 2019 ^15^ .

As variáveis antropométricas incluíram o IMC, CC, RCQ, relação cinturaestatura (RCE) e IC. Em todas as avaliações, foram seguidos os protocolos preconizados pelo *American College of Sports Medicine* ^16^ , todas em duplicata, sendo consideradas as médias das aferições em cada variável.

O peso foi mensurado por meio de uma balança digital Bal Gl 200 da G-Tech ^®^ com resolução de 50 gramas disposta em superfície plana. Os entrevistados foram orientados a usar roupas leves e convidados a subir descalços e com os bolsos vazios no centro da base da balança, com o corpo ereto e peso distribuído uniformemente entre os dois pés, braços ao lado do corpo e olhando para frente.

A estatura dos participantes foi determinada por um estadiômetro portátil Sanny ^®^ , com resolução em milímetros e a base disposta sempre sobre uma superfície plana. O participante, sem utilizar objetos na cabeça, foi disposto de costas para o antropômetro, com pernas e pés paralelos, peso distribuído em ambos, braços lateralizados e palmas das mãos voltadas para o corpo. Após o alinhamento da parte de trás da cabeça, costas, nádegas, pernas e calcanhares e olhar para frente utilizando o plano de Frankfurt para o posicionamento da cabeça, era solicitado ao entrevistado inspirar profundamente e prender a respiração durante a aferição, realizada deslocando a parte móvel do estadiômetro até o ponto mais alto da cabeça, comprimindo os cabelos o suficiente para obter a mensuração da estatura.

O IMC foi determinado pela razão da massa corporal em quilogramas pelo quadrado da estatura em metros. Foi adotada a seguinte classificação para adultos: eutrófico (< 25 kg/m ^2^ ), sobrepeso (25,0 a 29,9 kg/m ^2^ ) e obeso (≥ 30 kg/m ^2^ ) ^4^ . Para os idosos, a classificação foi: baixo peso (< 22 kg/m ^2^ ), eutrófico (22 27 kg/m ^2^ e sobrepeso (≥ 27 kg/m ^2^ ) ^17^ .

Foi utilizada uma fita inelástica Cescorf ^®^ com resolução em milímetros para a medida da CC, mensurada no ponto médio entre a crista ilíaca anterior superior e a última costela, com os participantes respirando normalmente e abdômen relaxado. A CC foi considerada normal quando menor que 102 cm em homens e menor que 88 cm em mulheres ^4^ . Para a medida do quadril, foi considerada a maior região de protuberância glútea no plano horizontal, com os participantes com os braços levemente afastados à frente e pés unidos, sendo a leitura da medida realizada na sua lateral. Ambas medidas foram utilizadas para o cálculo da relação cintura-quadril (RCQ = medida da cintura/medida do quadril), considerando-se adequados valores menores que 0,85 para as mulheres e menores que 0,90 para os homens ^18^ .

A relação cintura-estatura (RCE = medida da cintura/altura) foi considerada adequada quando menor que 0,5, conforme recomendação da Associação Brasileira para o Estudo da Obesidade e Síndrome Metabólica ^18^ . Para o cálculo do índice de conicidade, foi utilizada a equação:


$$IC\;=\;CC\;÷\;0,109\;\sqrt{\;peso\;÷\;altura}$$

A pressão arterial (PA), expressa em mmHg, foi obtida com um aparelho digital de aferição da pressão arterial de braço modelo BM35 da marca Beurer ^®^ . A PA foi aferida três vezes, uma após cinco minutos de repouso inicial e outras duas em intervalos de dois minutos, registrando-se a média, de acordo com as determinações das VI Diretrizes Brasileiras de Hipertensão Arterial. A hipertensão arterial foi definida como pressão arterial diastólica (PAD) maior ou igual a 90 mmHg e/ou pressão arterial sistólica (PAS) maior ou igual a 140 mmHg e/ou uso atual de medicação anti-hipertensiva ^20^ .

Para as análises laboratoriais das amostras de sangue, foi coletado sangue periférico da fossa antecubital, fracionado em dois tubos de ensaio para dosagem de triglicérides, colesterol total e frações (HDL, lipoproteína de alta densidade, e LDL, lipoproteína de baixa densidade) e glicemia, estando os participantes em jejum de 12h.

A presença de diabetes foi definida de acordo com os critérios da *American Diabetes Association* : glicose no plasma em jejum maior ou igual a 126 mg/dl, bem como a utilização de hipoglicemiante oral ou de insulina ^21^ . A dislipidemia foi definida pela presença de níveis anormais de um ou mais dos seguintes componentes lipídicos do sangue: colesterol total maior ou igual a 200 mg/dl, LDL maior ou igual a 160 mg/dl, triglicerídeos maiores ou iguais a 150 mg/dl e HDLmenor que 40 mg/dl em homens e menor que 50 mg/dl em mulheres, além do histórico de uso de medicamentos para redução desses valores ^3^ .

As análises estatísticas consistiram na distribuição das variáveis antropométricas segundo os fatores de risco cardiovascular por medidas de frequência e dispersão, segundo sexo e idade. Para avaliação da correlação das variáveis antropométricas com perfil lipídico, pressão arterial sistólica e diastólica e glicemia, foi utilizado o coeficiente de correlação de Pearson. Foram adotados níveis de significância de α < 0,05. A associação entre as variáveis antropométricas e as variáveis independentes entre os homens e mulheres foi realizada por meio das razões de prevalência (RP) e intervalos de confiança de 95% (IC95%). Em todas as análises, foram considerados o delineamento amostral e o peso das observações empregando as rotinas de *Complex Samples* do pacote estatístico SPSS ^®^ versão 20.0.

O presente estudo foi aprovado pelo Comitê de Ética em Pesquisa da Universidade Federal do Acre, sob o CAAE nº 17543013.0.0000.5010, tendo todos os participantes assinado o termo de consentimento livre e esclarecido.

## RESULTADOS

Nas avaliações das medidas de tendência central dos fatores de risco cardiovascular segundo as variáveis antropométricas, os homens adultos classificados com IMC ≥ 30 kg/m ^2^ , RCQ ≥ 0,90, CC ≥ 102, RCE > 0,5 e IC > 1,25 apresentaram médias alteradas de triglicerídeos (TG) e colesterol total (CT). Nas mulheres foram observadas médias acima dos valores de referência de TG para todos os indicadores antropométricos analisados e de CT entre aquelas com índice de conicidade alterado ( Tabela 1 ).

**Tabela 1:** Distribuição dos resultados dos exames laboratoriais indicadores de risco cardiovascular segundo variáveis antropométricas, por sexo, em adultos de Rio Branco, Acre. Brasil, 2014.

ADULTOS	HDL			LDL			CT			TG			GLI		
	M	EP	Md	M	EP	Md	M	EP	Md	M	EP	Md	M	EP	Md
Homens															
IMC (kg/m ^2^ )															
25	49.2	1.44	48.0	98.4	2.85	95.0	168.1	4.27	163.0	109.7	9.90	73.0	91.9	5.67	81.0
25–29.9	45.3	1.30	45.0	124.1	5.19	121.0	195.2	4.82	196.0	177.8	20.0	138.0	91.2	2.74	84.0
≥ 30	43.8	1.82	44.0	115.0	4.81	116.0	203.9	5.30	200.0	233.2	19.3	211.0	99.6	7.06	93.0
CC (cm)															
< 102	47.0	1.10	46.0	109.7	2.05	101.0	181.4	3.28	174.0	145.8	9.80	112.0	91.7	3.26	82.0
≥ 102	46.5	3.23	45.0	114.3	4.59	116.0	207.0	5.61	205.0	246.0	24.9	215.0	104.4	7.71	97.0
RCQ															
< 0.90	45.9	1.11	47.0	102.0	2.46	96.0	169.3	3.08	165.0	108.5	7.18	87.0	86.5	4.60	82.0
≥ 0.90	48.4	2.02	45.0	121.6	4.31	118.0	203.8	4.68	202.0	220.0	16.9	158.0	101.7	4.60	88.0
RCE															
< 0.5	46.1	1.09	96.0	98.0	2.77	94.0	163.8	3.51	160.0	100.5	7.93	76.0	86.8	5.25	81.0
≥ 0.5	47.7	1.83	113.0	121.3	3.61	118.0	201.9	3.69	202.0	204.6	14.4	153.0	98.4	3.86	87.0
IC															
< 1.25	45.6	1.03	44.0	104.2	2.00	99.0	175.0	2.79	170.0	127.5	8.76	108.0	87.8	3.66	82.0
≥ 1.25	50.4	2.86	47.0	125.6	5.29	117.0	200.8	4.70	205.0	225.5	24.6	153.0	105.9	5.71	91.0
Mulheres															
IMC (kg/m ^2^ )															
< 25	51.8	0.87	50.0	105.0	2.61	98.0	176.3	3.24	169.0	104.5	5.95	80.0	81.3	0.93	80.0
25–29.9	51.5	0.90	50.0	112.4	2.66	109.0	191.9	3.35	188.0	145.8	9.63	118.0	86.9	3.84	83.0
≥ 30	49.3	0.90	49.0	114.4	3.18	113.0	192.6	3.73	190.0	151.2	8.44	131.0	90.8	2.29	85.0
CC (cm)															
< 88	51.6	0.54	50.0	107.6	1.84	102.0	181.6	2.24	175.0	114.2	3.98	95.0	81.8	0.85	80.0
≥ 88	50.1	1.13	49.0	115.9	3.36	113.0	196.7	4.53	192.0	171.2	13.5	138.0	94.6	4.91	86.0
RCQ															
< 0.85	51.2	0.63	50.0	107.2	2.13	82.0	180.8	2.73	175.0	113.0	4.69	99.0	82.3	0.89	81.0
≥ 0.85	51.1	1.11	49.0	117.3	3.35	91.0	199.0	4.66	192.0	175.5	14.6	138.0	93.6	5.06	84.0
RCE															
< 0.5	51.6	0.79	50.0	101.4	2.08	96.0	172.9	2.79	168.0	99.2	4.74	83.0	81.2	0.88	80.0
≥ 0.5	50.8	0.71	50.0	117.1	2.32	113.0	196.6	3.05	192.0	156.1	8.19	129.0	88.9	2.69	84.0
IC															
< 1.18	50.8	0.60	49.0	105.7	1.81	102.0	175.0	2.79	173.0	109.5	3.49	95.0	81.9	0.94	80.0
≥ 1.18	51.9	1.00	51.0	118.5	3.43	113.0	206.2	5.75	192.0	171.6	12.99	138.0	92.3	4.26	85.0

IMC: índice de massa corporal; CC: circunferência da cintura; RCQ: relação cintura-quadril; RCE: relação cintura-estatura; IC: índice de conicidade; HDL: lipoproteína de alta densidade; LDL: lipoproteína de baixa densidade; CT: colesterol total; TG: triglicerídeos; GLI: glicemia; EP: erro-padrão; M: média; Md: mediana

Entre os idosos, os níveis de triglicerídeos foram elevados nos indivíduos que apresentaram IMC, CC, RCQ, RCE e IC alterados, em ambos os sexos. Entre as mulheres, em todos indicadores alterados, observaram-se valores médios mais elevados de CT ( Tabela 2 ).

**Tabela 2:** Distribuição dos resultados dos exames laboratoriais indicadores de risco cardiovascular segundo variáveis antropométricas, por sexo, em idosos de Rio Branco, Acre. Brasil, 2014.

IDOSOS	HDL			LDL			CT			TG			GLI		
	M	EP	Md	M	EP	Md	M	EP	Md	M	EP	Md	M	EP	Md
Homens															
IMC (kg/m ^2^ )															
< 22	49.5	1.28	51.0	109.3	3.63	109.0	180.3	5.04	175.0	108.1	9.01	85.0	96.4	6.41	83.0
22–27	49.0	0.86	48.0	120.1	2.88	115.0	197.1	3.56	193.0	147.8	7.58	116.0	95.7	3.92	84.0
> 27	46.8	1.55	44.0	116.0	3.31	118.0	197.6	3.27	195.0	196.7	10.6	165.0	101.4	3.02	91.0
CC (cm)															
< 102	48.2	0.69	47.0	116.4	2.18	115.0	194.4	2.75	191.0	157.3	7.03	125.0	97.8	2.87	86.0
≥ 102	47.6	3.29	43.0	119.7	5.75	119.0	199.2	4.98	204.0	197.2	12.0	184.0	100.4	2.30	96.0
RCQ															
< 1.0	51.3	1.40	51.0	118.7	5.84	119.0	193.6	6.70	193.0	122.0	9.93	92.0	102.4	6.30	86.0
≥ 1.0	47.6	1.02	46.0	117.0	2.07	115.0	195.7	2.48	192.0	171.0	7.97	140.0	98.0	2.79	87.0
RCE															
< 0.5	50.7	1.41	51.0	109.1	4.03	109.0	181.9	5.62	179.0	111.4	9.50	94.0	101.2	6.26	84.0
≥ 0.5	47.7	0.92	46.0	118.2	2.08	117.0	197.1	2.41	195.0	171.5	7.58	140.0	97.9	2.80	87.0
IC															
< 1.25	49.7	1.12	48.0	115.4	3.45	117.0	191.4	4.38	187.0	134.5	8.61	110.0	98.9	3.80	86.0
≥ 1.25	47.5	1.04	46.0	117.5	2.23	115.0	196.7	2.58	194.0	175.2	8.48	143.0	98.0	2.92	97.0
Mulheres															
IMC (kg/m ^2^ )															
< 22	56.9	1.62	57.0	123.3	4.09	122.0	205.7	5.53	210.0	131.5	7.13	116.0	96.8	8.08	86.0
22–27	58.0	1.26	56.0	131.1	3.28	127.0	221.0	4.52	218.0	161.2	6.57	136.0	104.7	4.20	88.0
> 27	54.3	0.88	53.0	124.1	2.56	121.0	212.8	3.07	205.0	179.2	5.68	158.0	104.6	3.00	92.0
CC (cm)															
< 88	57.3	0.88	56.0	129.7	2.79	127.0	216.8	3.60	213.0	154.3	4.78	134.0	101.2	3.70	87.0
≥ 88	54.5	0.95	53.0	123.0	2.26	119.0	212.6	2.84	205.0	179.7	6.00	158.0	105.7	3.45	92.0
RCQ															
< 0.85	58.2	1.26	57.0	125.9	3.45	121.0	213.1	4.61	205.0	149.6	6.46	135.0	98.9	4.50	87.0
≥ 0.85	55.0	0.77	54.0	126.6	2.04	124.0	215.2	2.45	211.0	173.4	4.66	154.0	105.1	2.94	90.0
RCE															
< 0.5	57.8	1.94	57.0	125.6	5.39	122.0	211.1	6.75	210.0	135.0	6.45	120.0	103.3	9.84	87.0
≥ 0.5	55.6	0.67	55.0	126.5	1.88	123.0	215.2	2.35	210.0	171.5	4.39	151.0	103.4	2.45	90.0
IC															
< 1.18	57.9	1.09	55.0	129.1	3.91	123.0	216.6	5.49	211.0	155.0	8.50	135.0	101.9	6.54	87.0
≥ 1.18	55.4	0.72	55.0	125.7	2.01	122.0	214.2	2.40	210.0	169.8	3.93	150.0	103.8	2.57	90.0

IMC: índice de massa corporal; CC: circunferência da cintura; RCQ: relação cintura-quadril; RCE: relação cintura-estatura; IC: índice de conicidade; HDL: lipoproteína de alta densidade; LDL: lipoproteína de baixa densidade; CT: colesterol total; TG: triglicerídeos; GLI: glicemia; EP: erro-padrão; M: média; Md: mediana

As maiores frequências de sobrepeso e obesidade nos adultos, segundo IMC, foram observadas na faixa etária de 40 a 59 anos: 39,5% e 22,6%, respectivamente, para os homens e 44,4% e 31,5% para as mulheres. Em relação à obesidade central, verificada pelos índices CC, RCQ e RCE, foram identificados maiores índices também na faixa etária de 40 a 59 anos, em ambos os sexos ( Tabela 3 ).

**Tabela 3:** Distribuição das variáveis antropométricas, segundo idade e sexo, em adultos e idosos de Rio Branco, Acre. Brasil, 2014.

	ADULTOS							IDOSOS					
	18–39 anos			40–59 anos				60–79 anos			80 anos e mais		
	n	N	%	n	N	%		n	N	%	n	N	%
IMC (kg/m ^2^ )							IMC (kg/m ^2^ )						
Homens							Homens						
< 25	48	36,327	51.6	38	11,821	37.9	< 22	31	879	9.4	19	401	26.0
25–29.9	37	25,405	36.0	38	12,318	39.5	22–27	148	4,256	45.5	33	695	45.0
≥ 30	13	8,711	12.4	22	7,041	22.6	> 27	140	4,216	45.1	21	448	29.0
Mulheres							Mulheres						
< 25	119	37,247	49.0	51	8,248	24.1	< 22	49	1,128	10.5	28	542	30.3
25–29.9	80	25,094	33.0	88	15,221	44.4	22–27	160	3,588	33.5	27	523	29.2
≥ 30	44	13,663	18.0	63	10,805	31.5	> 27	263	6,013	56.0	38	726	40.5
CC (cm)							CC (cm)						
Homens							Homens						
< 102	93	67,384	95.7	80	25,280	81.1	< 102	254	7,411	79.6	61	1,291	84.8
≥102	05	3,059	4.3	18	5,901	18.9	≥102	64	1,899	20.4	11	232	15.2
Mulheres							Mulheres						
< 88	190	59,501	78.6	114	19,190	56.0	< 88	213	4,851	45.4	52	1,003	56.0
≥ 88	52	16,197	21.4	88	15,084	44.0	≥ 88	257	5,832	54.6	41	788	44.0
RCQ							RCQ						
Homens							Homens						
< 0.90	66	49,096	69.7	31	9,865	31.6	< 0.90	38	1,098	11.9	10	216	14.2
≥ 0.90	32	21,347	30.3	67	21,316	68.4	≥ 0.90	278	8,155	88.1	62	1,307	85.8
Mulheres							Mulheres						
< 0.85	199	62,699	82.3	100	17,697	51.6	< 0.85	136	3,131	29.4	19	363	20.5
≥ 0.85	43	13,399	17.7	102	16,577	48.4	≥ 0.85	333	7,528	70.6	73	1,406	79.5
RCE							RCE						
Homens							Homens						
< 0.5	56	42,657	60.6	19	5,946	19.1	< 0.5	38	1,108	11.9	07	150	9.9
< 0.5	42	27,786	39.4	79	25,235	80.9	< 0.5	280	8,202	88.1	65	1,372	90.1
Mulheres							Mulheres						
< 0.5	134	42,082	55.6	47	8,108	23.7	< 0.5	56	1,292	12.1	10	190	10.6
≥ 0.5	108	33,616	44.4	155	26,166	76.3	> 0.5	414	9,391	87.9	83	1,601	89.4
IC							IC						
Homens							Homens						
< 1.25	81	59,007	83.8	44	14,493	46.5	< 1.25	87	2,614	28.1	11	238	15.7
≥ 1.25	17	11,436	16.2	54	16,688	53.5	≥ 1.25	231	6,696	71.9	61	1,284	84.3
Mulheres							Mulheres						
< 1.18	191	59,715	78.9	80	13,892	40.5	< 1.18	96	2,224	20.8	7	134	7.5
≥ 1.18	51	15,983	21.1	122	20,382	59.5	≥ 1.18	374	8,459	79.2	86	1,657	92.5

IMC: índice de massa corporal; CC:circunferência da cintura; RCQ: relação cintura-quadril; RCE:relação estatura; IC: Índice de conicidade; N:n expandido para a população.

Entre os idosos, as maiores frequências de excesso de peso pelo IMC foram observadas na faixa de 60 a 79 anos (45,1% para os homens e 56,0% para as mulheres). Nas mulheres de 80 anos ou mais também foi observada elevada frequência de excesso de peso. Ao analisar a obesidade centralnessa mesma população segundo a RCQ e a RCE, foram obtidas frequências acima de 70% nas duas faixas etárias, em ambos os sexos ( Tabela 3 ).

As variáveis antropométricas mostraram correlações estatisticamente significativas com variáveis do perfil lipídico e a glicemia; contudo, não passaram de correlações moderadas, sendo os mais expressivos em adultos do sexo masculino os coeficientes de correlação de RCQ, CC e RCE com triglicerídeos (r = 0,484, r = 0,438 e r = 0,448, respectivamente) e com o colesterol total (r = 0,486, r = 0,445 e r = 0,475, respectivamente). Nas mulheres adultas as maiores correlações observadas foram as da RCQ com o colesterol total (r = 0,350) e triglicerídeos (r = 0,345) e da RCE com pressão arterial sistólica (r = 0,369). Já para os idosos, a maior correlação estatisticamente significativa foi observada no sexo masculino, entre triglicerídeos e IMC (r = 0,251), conforme observado na Tabela 4.

**Table 4:** Matriz de correlação entre as variáveis antropométricas, perfil lipídico, glicemia e pressão arterial segundo o sexo em adultos e idosos de Rio Branco, Acre. Brasil, 2014.

	HDL	LDL	TG	CT	GLI	PAS	PAD
ADULTOS							
Mulheres							
IMC	-0.112 ^a^	0.100 ^a^	0.259 ^a^	0.147 ^a^	0.161 ^a^	0.288 ^a^	0.252 ^a^
CC	-0.079 ^a^	0.141 ^a^	0.33 ^6^ a	0.204 ^a^	0.210 ^a^	0.321 ^a^	0.275 ^a^
RCQ	-0.040 ^a^	0.115 ^a^	0.345 ^a^	0.35 ^0^ a	0.246 ^a^	0.337 ^a^	0.316 ^a^
RCE	-0.043 ^b^	0.149 ^b^	0.336	0.226 ^b^	0.196 ^b^	0.369 ^b^	0.321 ^a^
IC	-0.026 ^a^	0.005	-0.022 ^a^	-0.010 ^a^	-0.007 ^b^	-0.003	0.031 ^a^
Homens							
IMC	-0.145 ^a^	0.241 ^a^	0.37 ^7^ a	0.36 ^8^ a	-0.022 ^a^	0.243 ^a^	0.203 ^a^
CC	-0.026 ^a^	0.269 ^a^	0.43 ^8^ a	0.44 ^5^ a	0.047 ^a^	0.401 ^a^	0.350 ^a^
RCQ	0.027 ^a^	0.286 ^a^	0.48 ^4^ a	0.48 ^6^ a	0.142 ^a^	0.340 ^a^	0.371 ^b^
RCE	-0.036 ^a^	0.300 ^a^	0.448 ^a^	0.475 ^a^	0.096 ^a^	0.377 ^a^	0.351 ^a^
IC	0.135 ^a^	0.201 ^a^	0.324 ^a^	0.369 ^a^	0.161 ^a^	0.422 ^a^	0.404 ^a^
IDOSOS							
Mulheres							
IMC	-0.110 ^a^	-0.044 ^a^	0.102 ^a^	-0.021 ^b^	0.034 ^a^	0.051 ^a^	0.140 ^a^
CC	-0.039 ^a^	-0.037 ^a^	-0.038 ^a^	-0.055 ^a^	0.051 ^a^	0.064 ^a^	-0.010
RCQ	-0.149 ^a^	-0.024 ^a^	0.174 ^a^	0.077 ^a^	0.077 ^a^	0.124 ^a^	0.151 ^a^
RCE	-0.129 ^b^	-0.079 ^a^	0.114 ^b^	-0.044 ^a^	0.053 ^a^	0.109 ^a^	0.176 ^a^
IC	-0.038 ^a^	-0.037 ^a^	-0.039 ^a^	-0.055 ^a^	0.051 ^a^	0.064 ^a^	-0.010
Homens							
IMC	-0.088 ^a^	0.056 ^a^	0.251 ^a^	-0.110 ^a^	0.052 ^a^	0.174 ^a^	0.162 ^a^
CC	0.056 ^a^	0.028 ^a^	-0.051 ^a^	0.020 ^a^	-0.029 ^a^	-0.014	-0.025 ^a^
RCQ	-0.063 ^a^	0.005	0.073 ^a^	-0.007	0.000	0.130 ^a^	0.068 ^a^
RCE	-0.082 ^a^	0.050 ^ba^	0.219 ^a^	0.089 ^a^	0.034 ^a^	0.148 ^a^	0.156 ^a^
IC	0.056 ^a^	0.027 ^a^	-0.052 ^a^	0.019 ^b^	-0.029 ^a^	-0.014	-0.025 ^a^

IMC: índice de massa corporal; CC: circunferência da cintura; RCQ: relação cintura-quadril; RCE: relação cintura-estatura; IC: índice de conicidade; HDLlipoproteína de alta densidade; LDL: lipoproteína de baixa densidade; TG: triglicerídeos; CT:colesterol total; GLI: glicemia; PAS: pressão arterial sistólica; PAD: pressão arterial diastólica

^a^ p<0.01

^b^ p<0.05

As prevalências de hipertensão arterial foram maiores nos homens com obesidade segundo todos os indicadores analisados. Também no sexo masculino foram observadas as maiores prevalências de diabetes, a maior sendo para o indicador antropométrico IC (16,3%). Nos adultos de ambos os sexos, destacaram-se as prevalências acima de 78% de dislipidemia nos indivíduos obesos segundo todos os indicadores analisados (Tabela 5).

**Table 5:** Associação entre as variáveis antropométricas e os fatores de risco cardiovascular, segundo o sexo, em adultos de Rio Branco, Acre. Brasil, 2014.

	ADULTOS	HIPERTENSÃO		DIABETES		DISUPIDEMIA	
		Prevalência (%)	RP (IC95%)	Prevalência (%)	RP (IC95%)	Prevalência (%)	RP (IC95%)
Mulheres							
IMC (kg/m ^2^ )							
	25–29.9	14.6	2.32 (2.22–2.43)	2.2	2.34 (2.09–2.63)	78.7	1.16 (1.14–1.18)
	≥ 30	15.4	2.46 (2.34–2.58)	6.1	6.50 (5.84–7.24)	82.5	1.22 (1.20–1.24)
CC (cm)	≥ 88	19.8	2.51 (2.42–2.60)	6.6	7.10 (6.52–7.72)	81.8	1.13 (1.11–1.15)
RCQ	≥ 0.85	20.6	2.65 (2.56–2.74)	6.8	7.53 (6.92–8.20)	84.1	1.17 (1.15–1.19)
RCE	> 0.5	15.5	2.51 (2.41–2.62)	3.9	4.63 (4.17–5.13)	80.8	1.18 (1.16–1.20)
IC	≥ 1.18	20.3	2.96 (2.85–3.07)	6.0	7.39 (6.75–8.10)	81.1	1.12 (1.11–1.14)
Homens							
IMC (kg/m ^2^ )	25–29.9	14.8	2.19 (2.09–2.29)	8.1	1.47 (1.40–1.55)	78.1	2.04 (2.01–2.08)
	≥ 30	44.8	6.61 (6.34–6.89)	10.6	1.92 (1.81–2.04)	82.3	2.15 (2.11–2.20)
CC (cm)	≥ 102	63.3	5.74 (5.56–5.93)	8.9	1.27 (1.17–1.36)	80.9	1.39 (1.36–1.43)
RCQ	≥ 0.90	30.7	6.08 (5.84–6.32)	15.3	9.79 (9.14–10.49)	80.7	1.78 (1.75–1.81)
RCE	> 0.5	28.1	13.42 (12.58–14.31)	12.2	6.41 (5.98–6.87)	79.4	2.03 (1.99–2.06)
IC	≥ 1.25	37.3	5.10 (4.93–5.27)	16.3	4.32 (4.12–4.53)	80.0	1.52 (1.49–1.54)
Total							
IMC (kg/m ^2^ )	25–29.9	14.7	2.25 (2.18–2.32)	5.1	1.55 (1.47–1.62)	78.4	1.49 (1.47–1.51)
	≥ 30	27.0	4.14 (4.01–4.27)	7.9	2.40 (2.28–2.52)	82.4	1.57 (1.54–1.59)
CC (cm)	≥ 88 ≥ 102	29.5	3.01 (3.01–3.15)	7.1	1.67 (1.60–1.74)	81.6	1.26 (1.25–1.28)
RCQ	≥ 0.85 ≥ 0.90	26.5	4.00 (3.90–4.10)	11.8	9.92 (9.41–10.46)	82.1	1.36 (1.34–1.37)
RCE	> 0.5	21.4	5.13 (4.96–5.30)	7.8	5.71 (5.39–6.04)	80.1	1.48 (1.47–1.50)
IC	≥ 1.18 ≥ 1.25	27.8	3.92 (3.82–4.01)	10.5	4.57 (4.38–4.76)	80.6	1.29 (1.28–1.31)
Mulheres							
IMC (kg/m ^2^ )	> 27	71.5	1.65 (1.61–1.69)	20.8	1.15 (1.06–1.09)	87.3	1.05 (1.03–1.07)
CC (cm)	≥ 88	70.6	1.15 (1.13–1.66)	20.1	1.08 (1.06–1.09)	87.8	1.05 (1.03–1.06)
RCQ	≥ 0.85	68.3	1.16 (1.14–1.18)	18.2	1.06 (1.05–1.08)	88.7	1.12 (1.01–1.13)
RCE	> 0.5	66.5	1.21 (1.18–1.24)	17.4	1.07 (1.05–1.09)	86.0	1.02 (1.00–1.04)
IC	≥ 1.18	65.4	1.07 (1.04–1.09)	17.5	1.05 (1.03–1.07)	86.8	1.06 (1.04–1.07)
Homens							
IMC (kg/m ^2^ )	> 27	77.8	1.57 (1.53–1.61)	19.2	1.12 (1.10–1.15)	83.0	1.42 (1.38–1.46)
CC (cm)	≥ 102	72.1	1.09 (1.06–1.11)	22.6	1.11 (1.09–1.13)	89.1	1.23 (1.20–1.25)
RCQ	≥ 0.90	67.7	1.18 (1.15–1.21)	14.2	0.99 (0.97–1.01)	75.7	1.23 (1.20–1.26)
RCE	> 0.5	67.4	1.18 (1.15–1.21)	14.5	1.03 (1.01–1.05)	75.9	1.32 (1.28–1.35)
IC	≥ 1.25	67.7	1.08 (1.06–1.11)	14.2	1.00 (0.99–1.02)	77.0	1.18 (1.16–1.20)
Total							
IMC (kg/m ^2^ )	> 27	74.1	1.30 (1.59–1.64)	20.2	1.14 (1.12–1.15)	85.5	1.13 (1.12–1.15)
CC (cm)	≥ 88 ≥ 102	70.9	1.10 (1.09–1.12)	20.7	1.09 (1.08–1.10)	88.1	1.15 (1.13–1.16)
RCQ	≥ 0.85 ≥ 0.90	68.0	1.16 (1.15–1.18)	16.2	1.03 (1.02–1.04)	82.0	1.11 (1.10–1.13)
RCE	> 0.5	64.8	1.20 (1.17–1.22)	16.1	1.05 (1.03–1.06)	81.3	1.15 (1.13–1.16)
IC	≥ 1.18 ≥ 1.25	66.4	1.07 (1.06–1.09)	16.1	1.03 (1.01–1.04)	82.5	1.20 (1.18–1.22)

IMC: índice de massa corporal; CC: circunferência da cintura; RCQ: relação cintura-quadril; RCE: relação cintura-estatura; IC: índice de conicidade; RP: razão de prevalências; IC95%: intervalo de confiança de 95%

Ao analisar as associações, constatou-se maior força de associação entre hipertensão arterial e RCE (RP = 13,42; IC95% 12,58–14,31) e com IMC > 30 (RP = 6,61; IC95% 6,34–6,89) nos homens adultos. Na análise para diabetes, a RCQ apresentou maior robustez na associação para mulheres (RP = 7,53; IC95% 6,92–8,20) e homens (RP = 9,79; IC95% 9,14–10,49), como apresentado na Tabela 5.

Entre os idosos foram observadas prevalências de hipertensão arterial e obesidade maiores que as encontradas no grupo de adultos. As prevalências de hipertensão ficaram acima de 65% para homens e mulheres. Para diabetes a maior prevalência foi de 20,8% entre aqueles com IMC > 27 nas mulheres e 22,6% para CC alterada nos homens. As prevalências de dislipidemia foram similares às observadas nos adultos (Tabela 5).

Na análise bivariada, entre os idosos observaram-se menores efeitos de associação entre os indicadores antropométricos e os desfechos do que entre adultos. A maior associação verificada foi entre IMC e hipertensão arterial (RP = 1,65; IC95% 1,61–1,69) nos idosos homens, embora as razões de prevalência tenham se mantido similares em ambos os sexos nessa faixa etária (Tabela 5).

## DISCUSSÃO

Entre os principais achados do presente estudo, podem-se citar as correlações entre RCQ, CC e RCE com TG e CT nos homens adultos. As maiores frequências de obesidade geral foram encontradas nos adultos de 40 a 59 anos e nos idosos de 60 a 69 anos. Os marcadores do perfil lipídico estavam elevados nos adultos de ambos os sexos com IMC, CC, RCQ, RCE e IC alterados.

Estudos epidemiológicos têm mostrado uma correlação clara entre a obesidade e os fatores de risco cardiovascular ^22 , 23^ . Na presente análise, segundo a classificação por índices antropométricos, o sobrepeso e a obesidade foram frequentes. A pesquisa de Vigilância de Fatores de Risco e Proteção para Doenças Crônicas por Inquérito Telefônico (Vigitel) mostrou que 53,8% da população brasileira maior de 18 anos apresentava algum grau de excesso de peso, sendo Rio Branco a capital com a maior prevalência (60,6%) ^24^ .

Estudos internacionais ^2 , 25^ e nacionais ^23 , 24^ têm revelado elevada prevalência de excesso de peso, fenômeno mundialmente conhecido como transição nutricional que tem as alterações no padrão alimentar e de atividade física como fatores determinantes ^2 , 4^ . Atualmente é uma epidemia mundial, que atinge todas as faixas etárias, diferentes grupos socioeconômicos e países, causando inúmeros agravos e tornando esse distúrbio nutricional prejudicial à saúde pública ^4^ .

Na presente investigação, foi observado que o colesterol total (CT) e triglicerídeos (TG) estiveram mais correlacionados com a CC, RCQ e RCE no sexo masculino. Resultados semelhantes foram encontrados em estudo iraniano, que constatou correlações de CT e TG com a maioria dos índices antropométricos, especialmente nos homens ^22^ .

Embora o IMC seja habitualmente utilizado no rastreamento de obesidade, as medidas abdominais estão sendo amplamente empregadas e relacionadas na predição dos fatores de risco para DCV ^12^ . Em parte, isso decorre da observação de que a gordura abdominal está relacionada a várias anormalidades metabólicas, incluindo adversidades do perfil lipídico ^12^ .

No presente estudo, as prevalências de dislipidemia ficaram acima de 75%, independentemente do sexo, idade e indicador antropométrico analisado. Em estudo de base populacional realizado em São Paulo, as prevalências de dislipidemias foram de 73,1% e 69,9% em adultos e idosos, respectivamente, com excesso de peso segundo o IMC, e de 70,4% e 64,2% segundo a CC aumentada ^26^ . Outro estudo de base populacional com idosos da região Sul do Brasil mostrou que 70% das mulheres obesas tinha hipercolesterolemia e 64% hipertrigliceridemia; em contrapartida, nos homens a prevalência foi de 38,9% e 50,0%, respectivamente ^23^ .

Os indivíduos obesos estão mais suscetíveis a desenvolver diabetes e, sobretudo quando a obesidade está centralizada na região abdominal, as repercussões negativas, tanto de ordem metabólica quanto cardiovascular, são mais expressivas ^10^ . Uma vez que a gordura visceral é pro-inflamatória, pode ser infiltrada por macrófagos que podem levar à disfunção endotelial e posterior resistência à insulina ^7^ .

Estudo com a população idosa em São Paulo identificou a prevalência de diabetes de 21,3% entre aqueles com IMC > 27, semelhante à observada no presente estudo: 20,2% na população de idosos ^27^ . Embora os idosos tenham apresentado maiores prevalências em relação às observadas entre os adultos, as razões de probabilidade foram mais fortes entre os adultos. Uma possível explicação repousaria no viés de sobrevivência, uma vez que aqueles mais vulneráveis às complicações geradas pela doença teriam maior probabilidade de morrer prematuramente ^28^ .

Nos adultos, o indicador RCQ teve a associação mais forte com diabetes em ambos os sexos. Um estudo de revisão identificou a RCQ e a CC como os melhores preditores dos fatores de risco para doenças cardiovasculares ^10^ .

No presente estudo, as maiores frequências de obesidade geral e central em idosos foram observadas na faixa de 60 a 79 anos em ambos os sexos. Tendências atuais indicam que a prevalência nessa faixa aumentará, mesmo entre os grupos mais velhos. No estudo *Scottish Health Survey* , realizado entre os anos de 1998 e 2008, a obesidade verificada pelo IMC continuou a aumentar mesmo entre os indivíduos de 60 a 70 anos. No mesmo período observou-se um aumento de 5 a 10 cm da circunferência da cintura em ambos os sexos na faixa de 50 a 70 anos ^25^ .

O excesso de peso mostra-se associado à hipertensão arterial ^20 , 29^ . Esse fato pode ser explicado por alterações fisiológicas como ativação do sistema nervoso simpático e do sistema renina-angiotensina-aldosterona, resistência à insulina e disfunção renal e endotelial ^30^ .

No presente estudo foram observadas nos adultos prevalências de hipertensão arterial mais elevadas no sexo masculino (44,8%) que no feminino (15,4%) nos indivíduos obesos segundo o IMC. Dados similares foram encontrados em estudo de base populacional realizado em São Luís (MA), no qual 32,1% dos homens e 24,2% das mulheres eram hipertensos ^29^ . As VI Diretrizes Brasileiras de Hipertensão Arterial apontam que a prevalência global de hipertensão arterial entre homens é mais elevada nos homens até os 50 anos, invertendo-se a partir da quinta década ^20^ .

A obesidade apresentou-se como fator de risco para a hipertensão arterial, pois os indivíduos de ambos os sexos considerados obesos segundo o IMC e a RCE apresentaram um aumento de 6 a 13 vezes o risco de ter hipertensão, respectivamente. Também foi observada uma relação proporcional entre a prevalência de hipertensão arterial e o aumento de CC e RCQ, especialmente nos homens. Em estudo com indivíduos maiores de 18 anos, os indicadores IMC e CC foram considerados bons preditores do risco de desenvolver hipertensão arterial ^29^ .

Nos idosos foram encontradas prevalências de hipertensão arterial acima de 65% em homens e mulheres com excesso de peso e obesidade. O envelhecimento mostrou-se associado ao aumento da prevalência de HAS ^29^ em virtude da distensibilidade da aorta (complacência), redução do volume sistólico do ventrículo esquerdo e da velocidade de ejeção do ventrículo esquerdo ^31^ .

Algumas limitações podem ser reconhecidas neste estudo. Na análise dos resultados dos idosos, é necessária cautela em decorrência do viés de sobrevivência, tendo em vista que os fatores de risco para doenças cardiovascular levam ao óbito precocemente nos idosos acometidos por elas. Fazem-se necessárias outras análises longitudinais para fornecer evidências mais fortes das relações obtidas no presente estudo.

É importante ressaltar também que a coleta de amostras biológicas foi realizada em um único momento no tempo para definição de morbidades. No entanto, todas as análises foram realizadas em um mesmo laboratório para minimizar erros, sendo importante a utilização desses resultados a fim de obter maior fidedignidade nas definições de fatores de risco cardiovascular. Além disso, possíveis erros na verificação das medidas antropométricas foram minimizados pela duplicidade na verificação e a utilização das médias. Destaca-se também como um dos pontos fortes do presente estudo o fato de trabalhar com uma amostra de base populacional representativa de adultos e idosos da capital Rio Branco.

Os resultados obtidos mostram a relevância desses indicadores na identificação dos fatores de risco para as DCV e a importância de adotá-los na prática clínica e em estudos epidemiológicos com idosos e adultos, tendo em vista que são métodos simples, de baixo custo e não invasivos. Esses indicadores podem contribuir para a identificação precoce dos fatores de risco, possibilitando ações e estratégias de prevenção e controle das doenças cardiovasculares.
